# Some Day—A Midnight Reverie

**Published:** 1914-04

**Authors:** 


### Some Day—A Midnight Reverie.

Some day—the day of longings satisfied, of hopes realized, of ambitions gratified. Some day—the day when truth will prevail and error will cease to exist; when man will have faith in his brother and woman will believe her sister; when the child will not need to curse its begetter for its stigma of shame; when the father will not deny the child whose mother bore no gilt emblem of propriety upon the second finger of her left hand; when the universal conscience will decree that justice shall raise and exalt, not crush and destroy. Some day—when all will live in unison with each other, in harmony with the universe and its unalterable laws; when peace on earth, good will to men, will be real in the spirit and not a beautiful fancy. Some day—the day to come, be it the morrow or when the soul that moves the heart to beat and the brain to know leaves us as light leaves the candle which has been snuffed, and this mass of flesh and bone, brain and sinew becomes food for hungry worms or matter to follow the laws of chemical decomposition; or perhaps when today is reckoned in future ages, our mightiest efforts and our grandest deeds may become a study for wise men who want to know; what cheer, what promise in that some day, the red letter day of our lives or of eternity!

He who looks forward cares not to look back. Like to him who has crossed the river Lethe, the past is oblivion. If he looks beyond the six feet of earth and the marble slab that is destined to bear his name, to him is opened a vista, illimitable in its width, eternal in its length, glorious in its height, and filled with the wondrous works of man. And whether it be with the infinite intelligence that the great scientist tells us survives the grave, or whether it be with the eyes of the dreamer whose vision has no bounds, the view is the same; the same some day, the same man's mastery of time, of space, of life, of self. That some day man will learn the truth and then—*nirvana.*—Editorial from *American Practitioner,* New York, February, 1914.

---

An acutely appearing almond-sized or larger swelling in the skin of the submental region, which the patient usually thinks is an inflamed gland, is not an uncommon winter occurrence, especially in women, from exposure to the cold. No treatment is necessary other than protection of the part from further chilling. The prominences of the cheeks may also be thus affected, but less frequently. Sudden swelling and redness of the nose, frightening the patient into a diagnosis of erysipelas, is a less common "frost-bite" experience.—*American Journal of Surgery.*

